# Make it easier: 3-word strategies to help children with cerebral visual impairment use their vision more effectively

**DOI:** 10.1038/s41433-021-01920-4

**Published:** 2022-01-18

**Authors:** Rachel F. Pilling

**Affiliations:** School of Optometry and Vision Science, University of Bradford, Richmond Road, Bradford, BD7 1DP UK

**Keywords:** Vision disorders, Eye manifestations

## Abstract

**Introduction:**

Cerebral Visual Impairment (CVI) is the most common cause of visual impairment in children in the United Kingdom. Management relies on identifying strategies and adaptations which enable the child to use their vision effectively and efficiently. The majority of published strategies involve lengthy inventories used in a specialist setting. Feedback from parent support groups cite the provision of immediate advice on strategies at the time of diagnosis as a key indicator of good care. The aim of the study was to use the Delphi technique to construct a set of three-word phrases to succinctly describe strategies for common visual dysfunctions in children with CVI.

**Method:**

A panel of twelve experts across health, education and those with lived experience was recruited. Four rounds of questionnaires were used to reach consensus on candidates symptoms and suggestions for 3-word-phrases. Consensus was defined as 70% agreement.

**Results:**

Response rates were 92, 67, 92 and 91% for each round respectively. The 3-word phrases reaching consensus were: Big Bold Bright; Keep it Still; Eyes or Ears; Show It High; Better on Left/Right; Clear the Clutter; Keep It Short; Give Me Time, My Vision Varies, Just One Thing.

**Conclusion:**

The intention is for the phrases presented to act as a ‘starter’ at the point of diagnosis and are appropriate for children of any developmental or visual ability. The real-life validation of this set of expert-consensus phrases will require further studies, evaluating both their effectiveness in terms of mapping to an intervention and impact on visual development.

## Introduction

Cerebral Visual Impairment (CVI) is the most common cause of visual impairment in children in the United Kingdom [1]. CVI is defined as “a verifiable visual dysfunction not attributable to a disorder of the anterior visual pathways or co-occurring visual impairment[2]”. It is an umbrella term which encompasses the large number of visual processing difficulties which can occur in children, including difficulties with visual attention, visual field, visual perception, visual guided movement, shape, object or face recognition and sensory integration.

There has been a recent increase in interest in the identification and diagnosis of children with CVI. It is recognised that there is no cure for the condition; the mainstay of management is in identifying strategies which support the child, carer and teacher in understanding how the child uses their vision, and ways in which habilitation or adaptations can be made to enable the child to use their vision effectively and efficiently. Studies have shown that earlier intervention produces better improvement in visual function [3].

The provision of clear information to families and teachers has been noted as a key factor in bridging the gap between healthcare and education [4]. A paediatric ophthalmologist may be the first professional to convey the diagnosis to a parent or carer; as with any other medical condition, an explanation of the diagnosis along with what measures a parent can take to support their child is expected. There is a mismatch between the number of qualified teachers and the number of children in need. Furthermore, some services require a visual acuity threshold to be met or sight impairment registration before support is offered (personal communication).

Whilst it is beyond the scope of an ophthalmologist to construct specific educational requirements or interventions, Lehman suggests paediatric ophthalmologists should become comfortable and familiar with constructing global recommendations about how to support a child to more effectively use their vision that may impact on a child’s progress [5].

The Delphi technique has gained popularity as a method of gathering expert opinion on an area of interest. It promotes anonymity and avoids direct contact or confrontation between experts, and through reflection on feedback between rounds, “aids gradual formation of a considered opinion” [6]. In areas where creativity and diverse inputs are instrumental in developing novel strategies, a Delphi approach is commended.

The aim of the present study was to use a Delphi methodology to construct a set of three-word phrases which would succinctly describe the strategies which commonly apply to children with CVI, with the intention of supporting ophthalmologists and orthoptists provide immediate, easy to understand advice to families.

## Method

### Panel selection

The panel was recruited in two ways. Several of the panel were known to the author as experts in the field of cerebral visual impairment and were approached via email. In addition, panellists with lived experience of cerebral visual impairment were recruited via a twitter post by the author and through the social media channels of patient support groups for cerebral visual impairment (CVI Scotland and CVI Society). Participants were offered information about the study and asked to sign a consent form. All participants who returned the form by the date specified were included in the study. Ethics approval was granted by the Research Ethics Panel at the University of Bradford.

Questionnaires were developed using Microsoft Forms and emailed to participants.

All responses received within 2 weeks of the send date were included for analysis.

### Round 1

Round 1 comprised a short demographics page to determine the role of participants. A list of signs and symptoms described as visual behaviours associated with CVI were collated from reviews and text books [7–9]. Participants were asked to rank on a Likert scale (0 = unlikely to be useful; 1 = might be useful; 2 = likely to be useful; 3 = very likely to be useful) how useful a strategy for each symptom would be based on how often each was encountered in practice. An option to suggest other items was available; these would be included in Round 2 if suggested by two or more participants.

### Round 2

Items ranked 2 or 3 by at least 75% of participants in round 1 were combined to produce a short list of 10 items. These were presented as a list. Participants were asked then to provide a suggested 3-word phrase for their top five ranked options. An option to suggest other items was available. Items with 3-word phrases suggested by at least 75% of participants would be included in round 3.

### Round 3

Round 3 comprised all suggested 3-word phrases from round 2. Participants were asked to vote on which 3-word phrase they felt best represented a strategy to support each symptom, or suggest another option. Items reaching 70% agreement were considered as consensus.

### Round 4

Any items not reaching consensus in Round 3 were re-submitted to participants at Round 4. In keeping with Delphi methodology, the most popular response in Round 3 was clearly indicated, and how many participants had chosen this option. Any items not reaching consensus after Round 4 would be excluded from the final set of 3-word phrases.

## Results

### Round 1

Forms were sent to 12 participants and 11 replies were received (92% response rate). 5 (45%) participants were from education, 4 (36%) were eye health professionals and 2 (18%) were a parent or carer of a child with CVI. Table 1 shows the results. All items were scored 2 or 3 (likely or very likely to be useful) except for ‘seems to prefer other sensory stimulation instead of vision’, ‘problems understanding 3D world or making a mental map’ and ‘problems looking people in the eye’. No additional items were suggested.

**Table 1:** Symptoms and signs of visual dysfunction commonly seen in children with cerebral visual impairment.

**Poor vision/reduced visual acuity**
**Reduced contrast sensitivity**
**Unable to use one side of visual field/hemianopia**
**Difficulties using inferior visual field**
**Problems paying attention to more than one visual field at a time/hemifield inattention**
**Constricted visual field/narrow field of vision**
**Problems seeing moving objects/dyskinetopsia**
**Problems using vision to guide movement/optic ataxia**
**Problems seeing more than one object at once/simultagnosia**
Problems recognising faces/prosopagnosia
Problems understanding 3D world or making mental map
**Problems reading words/alexia**
**Problems recognising or using numbers to perform maths/dyscalculia**
Problems looking people in the eye
**Visual avoidance—deliberately looking away**
**Problems using vision and hearing at the same time**
**Problems maintaining visual attention for more than a short period**
**Visual fluctuates**
**Problems using vision while walking—bumps into things or needs to hold hands**
**Can only see things when they are moving**
**Tilts head or looks at objects out of the corner of their eye**
**Falls over clearly visible objects**
**Cannot find objects if they are in a pile or jumbled up**
Finds new places difficult to navigate
**Finds walking down steps/across uneven ground difficult**
**Needs lots of time to move head/eyes/hands/name an object**
**Finds it easier to see things using the edge of their vision rather than looking directly at them**
**Gets easily distracted by sounds, movement, other people**
**Seems to prefer other sensory stimulation (e.g., chewing, sucking, spinning, vocalising) instead of using vision**

Symptoms rated “likely” or “very likely” to be useful by 75% of participants are indicated in bold.

### Round 2

Items from round 1 were combined to create ten features (see Table 2). Forms were sent to 12 participants and 8 replies were received (67% response rate). Two participants contacted the author to explain they would abstain from this round. A mean of seven suggested phrases were made for each symptom (range 4–8).

**Table 2:** 3-word phrases suggested by panelists and results indicating most popular picks in Round 3 & 4. Phrases reaching consensus are highlighted.

Visual Symptom	3-word phrases suggested in Round 2	Selected by participants in Round 3	Selected by participants in Round 4
Cannot see moving objects	Keep it still	9	NA
	I cannot track	1	
	Slow it down	0	
	Other	1	
Cannot see and hear at the same time	Eyes or Ears	7	9
	See or Hear	3	1
	One sense only	1	0
	Keep it Quiet	0	NA
Cannot maintain visual attention	Keep it Short	5	9
	Keep it Simple	3	0
	Make it Quick	3	1
	Frequent breaks needed	0	NA
Cannot attend to more than one object at a time	Just one thing	6	10
	One object only	2	0
	One by One	2	0
	Show object individually	0	NA
	One or Other	0	NA
	Other	1	
Cannot use their inferior visual field	Show it High	5	8
	Raise it up	4	2
	Think object position	1	0
	Above shoulder height	1	0
	Show from above	0	NA
Cannot cope with visual clutter	Clear the clutter	5	9
	One thing only	2	0
	Use plain background	2	0
	Keep it simple	1	1
	Keep it tidy	1	NA
Cannot use both visual fields equally	Better on left/right	6	9
	Show on left/right	3	1
	On the left/right	1	0
	Turn body left/right	0	NA
	Other	1 “use right/left”	
Cannot maintain consistent vision	My vision varies	8	NA
	Allow rest time	1	
	Remember rest breaks	1	
	Eyes get tired	0	
	Vision up& down	0	
	Other	1 “I’m easily overwhelmed”	
Cannot immediately respond to objects	Give me time	6	9
	Wait for eight	3	1
	Slow the pace	1	0
	I need time	0	NA
	Processing time needed	0	NA
	Other	1 “It would depend on the cause”	
Cannot see small/low contrast	Big Bold Bright	10	NA
	Make it clear		
	Bigger is better		
	Keep it big		
	Other	1 comment on underlying mechanism	

### Round 3

Ten items for which 6 or more participants had suggested a 3-word phrase were included in round 3. Suggestions were presented verbatim. Forms were sent to 12 participants and 11 replies were received (92% response rate). Three items reached consensus. One participant chose to leave the study after this round.

### Round 4

The remaining seven items were presented to participants, including only those options which had received one or more votes in round 3. The most popular item from Round 3 was shown first in the list. Forms were sent to 11 participants and ten replies were received (91% response rate). All remaining items reached consensus.

The final list of 3-word phrases which can be used to describe strategies to support someone with cerebral visual impairment or brain based visual difficulties are presented in Table 3.

**Table 3:** Final list of 3-word phrases indicating strategies which support visual dysfunctions commonly seen in children with cerebral visual impairment.

Symptom/visual behaviour descriptor	3 word phrase
Right/Left HemiField inattention	Better on Left/Right
Inferior visual field inattention	Show It High
Dyskinetopsia (difficulty seeing moving objects)	Keep It Still
Simultagnosia (difficulty seeing more than one object)	Just One Thing
Sensory integration (difficulty seeing and hearing at the same time)	Eyes or Ears
Visuo-motor delay (delay in responding to visual objects)	Give Me Time
Difficulty maintaining visual attention	Keep It Short
Difficulty with busy visual scenes	Clear the Clutter
Variable visual function	My Vision Varies
Reduced visual acuity or contrast	Big Bold Bright

## Discussion

The James Lind Alliance has ranked the identification, prevention and treatment of cerebral visual impairment in children as its number one research priority. The pursuit of strategies to support children diagnosed with cerebral visual impairment or brain based visual difficulties has been the subject of several landmark publications, resulting in the widespread use of inventories or questionnaires, completed by parents or carers and mapping to a long list of options for families and educators to consider [8, 10–14]. The time taken to complete these inventories makes utilising such tools in a clinical environment challenging. The majority of published strategies are not appropriate for non-verbal, non-mobile children and it is this group who are most often seen in the paediatric ophthalmology clinic, commonly referred by paediatricians as part of the pathway for a child with developmental delay. It is clear from parent feedback that the provision of early information at the time of diagnosis including simple ways to help the child’s visual functioning would be seen as an improvement and have an impact on patient experience in the clinic.

The aim of the study was to produce a set of simple global strategies relating to the most commonly seen features of cerebral visual impairment in children of all academic abilities. The most frequently noted features of cerebral visual impairment were common to education, eye health professionals and parents/carers. A range of suggestions for common symptoms were suggested, and consensus was reached for all ten phrases. It is anticipated that eye health professionals and/or teachers of the visually impaired may identify visual dysfunction in each individual child, and use the phrases shown in Table 3 to provide one or two suggestions of simple strategies to parents, teachers and those involved with habilitation or therapies. A set of bright coloured icons are being developed for this purpose and will be made available through patient support group websites.

Generally response rates of 70% or higher are considered good in Delphi studies [15].

The response rate in this study was 92% in rounds 1 and 3, and 91% in round 4. Whilst the response rate in round 2 was lower than expected (67%), two respondents corresponded with the author to explain their lack of participation in this round, as they felt they had no suggestions of 3-word phrases to offer.

The current study is limited in its recruitment of small numbers from a diverse group of professionals. The panel size of 12, while small, lies within the recommended range by Turoff [16]. In studies where novel ideas are being sought, a small sample size makes data processing manageable. The strategy of purposive sampling, where individuals are approached and invited, rather than random selection, is a principle which underpins Delphi studies. In this study, there were few examples of divergence of opinion in early questionnaires; whilst this may represent areas of agreement between experts, it may also be due to a narrow field of opinion. However, the number of professionals in the UK with expertise in Cerebral Visual Impairment is small. All participants had over five years’ experience in the field and are seen as national experts. In order to minimise this bias, a balanced panel of professionals from eye health, education and patients/carers were recruited. A wider participant field might increase the risk of gaining responses from those who lacked real-world experience.

The Delphi approach is suitable for the generation of ideas from a diverse group in an area where a lack of clarity currently exists, and was ideally suited to this research question. Studies of this nature generate findings based purely on expert opinion. The anonymous nature of the feedback between rounds serves to reduce the chance of panellists deferring to an opinion based on the respondent’s job title or role, there being a tendency to agree with either those perceived as higher in a hierarchy, or being “like me”.

The formulation of an individual programme of educational adaptations can only be developed following in-depth educational or psychological assessment within the child’s usual environment. Such a report is beyond the remit of a paediatric ophthalmologist and referral to the visual impairment team to devise should follow a diagnosis of cerebral visual impairment. The intention is for the phrases presented from this study is to act as a “starter” at the point of diagnosis and are appropriate for children of any developmental or visual ability. The real-life validation of this set of expert-consensus phrases will require further studies, evaluating both their effectiveness in terms of mapping to an intervention and impact on visual development.

## Summary

### What was known before

- Cerebral Visual Impairment (CVI) is the most common cause childhood visual impairment in the UK.
- The mainstay of management of CVI is supporting parents and patients in learning how to use their vision effectively.

### What this study adds

- A set of 3-word phrases which indicate a simple strategy to support common visual dysfunctions seen in CVI.
- “Make it Easier” is the overriding theme in helping children with CVI and can be commended while awaiting an in-depth educational assessment.
- Ophthalmologists are encouraged to use this set of strategies to offer accessible advice at the time of diagnosis.
